# CARDIOMETABOLIC RISK FACTORS ASSOCIATED WITH ACTIVE COMMUTING TO SCHOOL

**DOI:** 10.1590/1984-0462/;2019;37;2;00007

**Published:** 2019-02-25

**Authors:** Miria Suzana Burgos, Debora Tornquist, Luciana Tornquist, Cézane Priscila Reuter, Edna Linhares Garcia, Jane Dagmar Pollo Renner, Andréia Rosane de Moura Valim

**Affiliations:** a Universidade de Santa Cruz do Sul, Santa Cruz do Sul, RS, Brazil.

**Keywords:** Risk factors, Physical activity, Child, Adolescent, Fatores de risco, Atividade física, Criança, Adolescente

## Abstract

**Objective:**

To verify if there is an association between cardiometabolic risk factors and active daily commuting to school among children and adolescents.

**Methods:**

A total of 1,743 schoolchildren aged 7 to 17 years old were evaluated in the city of Santa Cruz do Sul (RS). The way of commuting to school was investigated with a questionnaire, and the cardiometabolic risk factors analyzed were body mass index (BMI), waist circumference (WC), systolic (SBP) and diastolic (DBP) blood pressure, blood glucose, triglycerides, total cholesterol (TC) and fractions, LDL and HDL.

**Results:**

The prevalence of active commuting among schoolchildren was 48.0% (95%CI 45.7-50.4), and it was associated, in the crude analysis, with blood glucose and LDL cholesterol levels. Passive schoolchildren had a 1.1 higher prevalence ratio of high glucose and LDL cholesterol levels. However, when sociodemographic variables were included in the model, these associations were not maintained.

**Conclusions:**

The prevalence of active commuting in the sample studied is low and it was shown to have a crude association with glucose and LDL cholesterol levels in students. However, sociodemographic factors seem to influence these associations.

## RESUMO

**Objetivo:**

Verificar se existe associação entre fatores de risco cardiometabólicos e deslocamento ativo à escola em crianças e adolescentes.

**Métodos:**

Foram avaliados 1.743 escolares, de sete a 17 anos, do município de Santa Cruz do Sul (RS). A forma de deslocamento até a escola foi investigada por meio de questionário e os fatores de risco cardiometabólicos analisados foram: o índice de massa corpórea (IMC), a circunferência da cintura (CC), a pressão arterial sistólica (PAS) e a diastólica (PAD), glicose, triglicerídeos, colesterol total (CT), LDL e HDL.

**Resultados:**

A prevalência de deslocamento ativo entre os escolares foi de 48,0% (IC95% 45,7-50,4) e associou-se, na análise bruta, com os níveis de glicose e colesterol LDL. Escolares que se deslocavam de forma passiva apresentaram uma razão de prevalência (RP) 1,1 vez maior de glicose e colesterol LDL elevados. No entanto, ao serem incluídas variáveis sociodemográficas no modelo, essas associações não se mantiveram.

**Conclusões:**

Conclui-se que a prevalência de deslocamento ativo na amostra estudada é baixa e que o deslocamento ativo à escola apresentou associação bruta com os níveis sanguíneos de glicose e de colesterol LDL dos escolares, sendo que se deslocar de forma ativa parece auxiliar na redução desses níveis. Porém, fatores sociodemográficos parecem exercer influência sobre estas associações.

## INTRODUCTION

Insufficient levels of physical activity among children and adolescents have become increasingly prevalent across the globe, impacting the well-being and health of this population.^1^ Data from the National School Health Survey show that only 30,1% of Brazilian schoolchildren reach the recommended 300 weekly minutes of physical activity, while the South region reaches 36.3%.^2^

Active commuting to school, characterized by walking and/or pedaling to school, has been widely adopted as an effective strategy to increase the levels of daily physical activity of young people, helping to sustain an active lifestyle.^3^ This practice, however, can impact health positively and, consequently, help to prevent metabolic and cardiovascular diseases.^4^

Studies have shown a positive association of active commuting to work with physical fitness levels among adults^5^ and negative association with obesity indicators,^5^ ^.^ ^6^ triglyceride levels,^5^ blood pressure^5^ ^,^ ^6^ and insulin resistance indicators, besides being likend to an 11% reduction in cardiovascular risk.^7^

The national scientific literature brings studies conducted with Brazilian children and adolescents on the prevalence of active commuting to school and its association with socioeconomic factors^8^ ^,^ ^9^ ^,^ ^10^ ^,^ ^11^ ^,^ ^12^ and with free time activities,^11^ obesity^10^ ^,^ ^13^ and blood pressure indicators.^10^ However, there are no studies addressing the association of active commuting with other cardiometabolic risk factors. This study, therefore, aims to search and idetify any associations between cardiometabolic risk factors and active commuting to school among children and adolescents.

## METHOD

This is a cross-sectional study that has been developed from the research database of the study “Evaluation of biochemical health indicators of schoolchildren using infrared spectroscopy, polymorphisms, oral health and lifestyle factors: a study in Santa Cruz do South - Phase II”. The project was approved by the Research Ethics Committee of the Universidade de Santa Cruz do Sul (protocol 3044-11). Participating schools received a copy of the research project and had their policy boards signing an acceptance letter. All students evaluated had their parentes/caregivers signing the free and informed consent.

Thee Poisson regression was used as statistical test for sample calculation on G* Power 3.1 program (Heinrich-Heine-Universität - Düsseldorf, Germany), with test power (1-β) = 0.95, significance level of α = 0.05 and effect size of 0.30, as proposed by Faul et al.^14^ The prevalence of schoolchildren enrolled in all education networks in the city was assessed from data collected in 2007 at the 6th Regional Education Coordination and Municipal Education Secretariat of Santa Cruz do Sul, where it was found that the population consisted of 20,540 primary and secondary school students from 69 schools in both the public and private networks. A representative sample was estimated in approximately 400 subjects. However, the authors decided to extend and open the opportunity to a larger number of schoolchildren in the city.

An estimate of the number of students and schools per network, zone and region to compose the sample was made. Schools were randomly chosen from a sample stratified by clusters. To ensure the representativeness and proportionality of students by region, the municipality was divided into five geographic areas: Central, North (urban and rural), South (urban and rural), East (urban and rural), and West (urban and rural).

Data were collected in 2011 and 2012 twice a week at the university. The measures and evaluations were carried out by a previously trained team of evaluators (professors and scholarship holders/scholars from various courses in the field of health and from the Master’s in Health Promotion).

The participation in data collection at a school was scheduled weekly in advance by telefone, with the policy team. The participants of each school were chosen randomly, through the list of students’ name previously sent by the institution via e-mail. In all schools, at least one class of each year and stage of teaching was invited to participate. The free and informed consent form was sent for the selected students through the school prior to data collection, as well as a note to parentes, explaining the procedures of data collection.

Nineteen schools participated: seven from the municipal network, ten from the state and two from the private network; being 14 in the urban area and five in the rural area; ten schools had only elementary education and nine had both primary and secondary education. Inclusion criteria were: being aged 7 to 17 years old and not presenting intellectual or cognitive deficiencies or limitations in order to understand and fill in the research instrument, or any physical limitations that could interfere in other evaluations. Schoolchildren who did not fill in the research instruments correctly, who for some reason did not perform any of the evaluations or from which blood samples could not be collected were excluded.

Regarding socio-demographic characteristics, participants under the age of ten were considered children and those aged ten on were classified as adolescents, according to World Health Organization (WHO) criteria.^15^ The socioeconomic level was classified according to ABEP,^16^ which groups subjects into eight distinct economic classes (A1, A2, B1, B2, C1, C2, D and E). For the present study, these classes were regrouped in three: high economic classes (A1, A2, B1 and B2), intermediate classes (C1 and C2) and lower classes (D and E).

Information on how to get to school is part of a series of information contained in the survey completed by the schoolchildren during data collection. The questionnaire “Lifestyle, Health and Welfare - child/adolescente”, by Barros and Nahas,^17^ was adapted by the researchers to meet the objectives of the project. As for commute to school, the question was: “How do you predominantly go to school (college)?”, with the following options: bus; on foot; car or motorcycle; bike; other (please specify). Answers were classified into active (walking, cycling or other form of movement requiring physical effort) or passive means of transportation (including alternatives such as bus, car, motorcycle, school bus, and other types of motor vehicle).

Body mass index (BMI) was determined by measuring body mass and height of schoolchildren, with classification based on the criteria suggested by the Centers for Disease Control and Prevention,^18^ according to sex and age: low weigh (p<5), normal weight (p≥5 and p≥85), overweight (p≥85 and p<95) and obesity (p≥95). All four categories were grouped in two, that is, low/normal weight and overweight/obesity.

Waist circumference (WC) was measured using an inelastic metric tape with 1-mm resolution (Cardiomed^®^, Curitiba, Brazil), based on the narrower part of the trunk between the ribs and the iliac crest. Subsequently, the measure was classified according to criteria established by Taylor et al.,^19^ considering circumference normal (percentile ≤75) or high (percentile >75), according to gender and age.

Blood pressure was measured by auscultation, with the student sitting and in rest of five minutes. A Sphygmomanometers for brachial perimeter and a stethoscope were placed on their left arm. Each device had three different sized cuffs so that researchers could select the most suitable for each arm circumference (pediatric, adolescent, and adult). Blood pressure was sorted based on percentiles for age, sex and height, with percentile <90 considered normal, between 90 and 95 borderline, and above 95 hypertension (stages 1 and 2), according to the VI Brazilian Guidelines for Hypertension;^20^ these were then regrouped into two categories: normotensive and borderline/hypertensive.

In order to evaluate the biochemical indicators (blood glucose, triglycerides, total cholesterol - low density lipoprotein LDL and high-density lipoprotein HDL cholesterol), students in fasting and previous rest of 12 hours were submitted to standard blood collection from the brachial vein with *vacutainer* without serum additives. The blood samples were incubated at 37 °C for 15 minutes and then centrifuged at 2,500 rpm at the same time to obtain serum samples. these were subjected to glucose, triglycerides and HDL testing in the automated equipment Miura One^®^ (ISE, Rome, Italy) using commercial DiaSys^®^ kits (Diagnostic Systems, Germany). The Friedwald equation was used to determine LDL:^21^ LDL = CT-HDL- (Triglycerides/5).

CT, LDL, HDL and triglycerides were classified according to the National Heart, Lung, and Blood Institute^22^, considering categories borderline/increased for CT, LDL and triglycerides, and acceptable and borderline/low for HDL. The American Diabetes Association protocol was used for glucose measurement,^23^ sorted as normal (up to 99 mg/dL), pre-diabetes (100-126 mg/dL), and diabetes (≥126 mg/dL).

Statistical analysis was performed in the Statistical Package for the Social Sciences (SPSS^®^) version 20.0 (IBM, Armonk, USA). Descriptive statistics were initially used to analyze the distribution of frequency of variables and respective 95% confidence intervals (95%CI). The chi-square test was applied to analyze the distribution of frequency of means of commuting to school according to sociodemographic factors, with significance level set at p≤0.05. Poisson regression was applied to test the association of commuting to school with cardiovascular risk factors, while crude and adjusted prevalence ratios with respective 95%CIs were calculated to estimate the extent of the associations’ effect. For the adjusted analysis, sociodemographic variables (such as gender, age group, school network, region of residence, and economic class) were included in the model. The level of significance was 5%.

## RESULTS

In total, 1,963 students were evaluated. After applying the exclusion criteria, 220 (11.2%) participants were excluded from the sample due to data inconsistency, missing info or impossibility to collect blood samples. Thus, 1,743 students made up the final sample, of which 53.8% were females. Table 1 shows the sociodemographic characteristics of the sample and, Table 2, the frequency distributions of cardiometabolic risk factors.

**Table 1:** Sociodemographic characteristics of schoolchildren included in the sample.

	n	% (95%CI)
Gender		
Males	806	46.2 (43.9-48.5)
Females	937	53.8 (51.5-56.1)
Age group		
Children (aged 7-9)	454	26.0 (24.0-28.1)
Adolescents (aged 10-17)	1.289	74.0 (71.9-76.0)
School network		
Municipal	760	43.6 (41.1-46.1)
State	889	51.0 (48.5-53.4)
Private	94	5.4 (4.4-6.5)
Region		
Central	343	19.7 (17.8-21.7)
Periphery	768	44.0 (41.8-46.4)
Rural	632	36.3 (34.0-38.5)
Economic class		
A and B	828	47.5 (45.2-49.8)
C	844	48.4 (46.1-50.9)
D and E	71	4.1 (3.2-5.0)

N: sample size; IC95%: 95% confidence interval.

**Table 2:** Characterization of way of commuting and cardiometabolic risk factors of schoolchildren included in the sample (n = 1,743).

	n	% (95%CI)
Commuting to school		
Active	837	48,0 (45,7-50,4)
Passive	906	52,0 (49,6-54,3)
BMI		
Low/normal weight	1,236	70.9 (68.8-73.1)
Overweight/obesity	507	29.1 (26.9-31.2)
WC		
Normal	1,400	80.3 (78.4-82.3)
Increased	343	19.7 (17.7-21.6)
SBP		
Normal	1,507	86.5 (84.9-88.1)
Borderline/hypertension	236	13.5 (11.9-15.1)
DBP		
Normal	1,477	84.7 (83.2-86.4)
Borderline/hypertension	266	15.3 (13.6-16.8)
Glucose levels		
Acceptable	1,430	82.0 (80.1-83.8)
Pre-diabetes/diabetes	313	18.0 (16.2-19.9)
TG		
Acceptable	900	51.6 (49.3-53.9)
Borderline/increased	843	48.4 (46.1-50.7)
TC		
Acceptable	684	39.2 (37.1-41.5)
Borderline/increased	1,059	60.8 (58.5-62.9)
LDL		
Acceptable	991	56.9 (54.6-59.2)
Borderline/increased	752	43.1 (40.8-45.4)
HDL		
Acceptable	1,457	83.6 (81.8-85.3)
Borderline/low	286	16.4 (14.7-18.2)

N: sample size; IC95%: 95% confidence interval; BMI: body mass index; WC: waist circumference; SBP: Systolic blood pressure; DBP: Diastolic blood pressure; TG: Triglycerides; TC: Total cholesterol; LDL: Low-density lipoprotein; HDL: High density lipoprotein.

The prevalence of active commuting to school among schoolchildren was 48.0% (95%CI 45.7-50.4), being higher for males (p=0.031), children aged seven to nine years (p=0.009), students of the State school network, living in the periphery, and from lower economic classes (p<0.001), according to data listed in Table 3.

**Table 3:** Frequency distribution of way of commuting to school according to sociodemographic factors (n=1,743).

	Active commuting		Passive commuting		p-value*
	n	% (95%CI)	n	% (95%CI)	
Gender					
Male	407	50.5 (46.9-53.9)	399	49.5 (46.1-53.1)	0.031
Female	430	45.9 (42.7-49.1)	507	54.1 (50.9-57.3)	
Age group					
Children (aged 7-9)	240	52.9 (48.4-57.9)	214	47.1 (42.1-51.6)	0.009
Adolescents (aged 10-17)	597	46.3 (43.8-49.1)	692	53.7 (50.9-56.2)	
School network					
Municipal	338	44.5 (41.1-47.9)	422	55.5 (52.1-58.9)	<0.001
State	485	54.6 (51.3-57.7)	404	45.4 (42.3-48.7)	
Private	14	14.9 (8.5-22.3)	80	85.1 (77.7-91.5)	
Region					
Central	112	32.7 (27.7-37.6)	231	67.3 (62.4-72.3)	<0.001
Periphery	636	82.8 (80.2-85.4)	132	17.2 (14.6-19.8)	
Rural	89	14.1 (11.6-16.9)	543	85.9 (83.1-88.4)	
Economic class					
A and B	345	41.7 (38.5-45.0)	483	58.3 (55.0-61.5)	<0.001
C	454	53.8 (50.4-57.3)	390	46.2 (42.7-49.6)	
D and E	38	53.5 (42.3-65.3)	33	46.5 (34.7-57.7)	

N: sample size; IC95%: 95% confidence interval. *Chi-square test, with statistical difference at p≤0,05.

In the crude analysis, active commuting was associated with risk factors for glucose and LDL cholesterol. Schoolchildren who go to school passively have 1.1 higher prevalence ratio (PR) for glucose levels (p=1.10, 95%CI 1.01-1.20) and high LDL cholesterol levels (PR 1,12; 95%CI 1.03-1.21). However, when adjusted for socioeconomic variables, these associations were not maintained (Table 4).

**Table 4:** Ratio of crude and adjusted prevalence of cardiometabolic risk factors related to school commuting among students included in the sample (n = 1,743).

Way of commuting	Crude PR (95%CI)	p-value	Adjusted PR* (95%CI)	p-value
BMI				
Active	1	0.485	1	0.509
Passive	0.97 (0.89-1.06)		0.97 (0.89-1.06)	
WC				
Active	1	0.885	1	0.816
Passive	0.99 (0.91-1.08)		0.99 (0.90-1.09)	
SBP				
Active	1	0.380	1	0.690
Passive	1.04 (0.96-1.13)		1.02 (0.93-1.11)	
DBP				
Active	1	0.691	1	0.863
Passive	1.02 (0.94-1.10)		1.01 (0.92-1.10)	
Glucose levels				
Active	1	0.033	1	0.407
Passive	1.10 (1.01-1.20)		1.04 (0.95-1.14)	
TG				
Active	1	0.220	1	0.363
Passive	0.96 (0.89-1.03)		1.04 (0.96-1.13)	
TC				
Active	1	0.127	1	0.218
Passive	1.06 (0.98-1.15)		0.95 (0.88-1.03)	
LDL				
Active	1	0.006	1	0.375
Passive	1.12 (1.03-1.21)		1.04 (0.96-1.13)	
HDL				
Active	1	0.906	1	0.731
Passive	0.99 (0.91-1.09)		0.98 (0.90-1.08)	

N: sample size; IC95%: 95% confidence interval; BMI: body mass index; WC: waist circumference; SBP: Systolic blood pressure; DBP: Diastolic blood pressure; TG: Triglycerides; TC: Total cholesterol; LDL: Low-density lipoprotein; HDL: High density lipoprotein. *Adjusted for gender, age group, school network, region and economic class.

## DISCUSSION

The main finding of this study is the association of passive commuting with glucose and LDL cholesterol levels: in the crude analysis, schoolchildren who passively commute to school have a 1.1 higher PR of increased levels. However, after analysis adjusted for socioeconomic factors, this relationship is not maintained.

The habit of actively commuting daily to school is an important source of daily activity and a relevant increase in physical activity levels.^3^ The effects of good levels of physical activity on glucose and lipid metabolism are well-evidenced already. It is known that exercise creates hormonal changes that activate the translocation of glucose transporter 4 (GLUT4) and thus increase glucose uptake regardless of the action of insulin.^24^ Physical exercise also helps increase glucose enzymatic activity in lipid metabolism, increasing the catabolism of triglycerides, while reducing the formation of LDL particles and increasing the production of HDL.^25^

Regarding the non-association between the way of commuting and anthropometric indicators, Heelan et al.^23^ have pointed out that active commuting does not seem to provide a sufficient amount of physical activity to reduce these effects, although it does help to raise the levels of this activity. On the non-association with blood pressure levels, Silva and Lopes^10^ have stated that the relation between blood pressure and physical activity in this age group has not been well established yet, and cross-sectional epidemiological studies do not allow observing cause and effect between such variables.

According to a previous literature review, other studies evaluating the association between commuting to school and the same cardiometabolic risk factors analyzed in this study in the Brazilian schoolchildren population are unknown. However, some studies address the association with some of the variables considered in this project. A study conducted with schoolchildren from João Pessoa, Paraíba, to evaluate the association of passive commuting with BMI, fat percentage and blood pressure reported association only with anthropometric indicators, BMI and fat percentage.^10^ This association with BMI was also investigated and seen among schoolchildren from Uruguaiana, Rio Grande do Sul,^13^ and Florianópolis, Santa Catarina.^26^ Among schoolchildren from Caxias do Sul, Rio Grande do Sul, it sedentary commuting to school was shown to increase the chance of high TC.^27^

A study with Portuguese schoolchildren found a greater probability of students who actively commute to present better indexes for WC and HDL cholesterol than sedentary ones.^4^ A longitudinal study conducted in Denmark followed 334 children over six year and showed that those who used the bicycle as a means of transportation to school had a better cardiovascular risk profile. Students who, over time, started cycling to school, showed improved HDL cholesterol and glucose levels, as well as a reduced cardiovascular risk levels compared to those who did not use the bycicle.^28^

In this study, the crude effect of commuting to school on glucose and LDL cholesterol levels was not confirmed in the analysis adjusted for sociodemographic variables. This loss of significance after adjustment indicates that glucose and LDL levels are influenced by sociodemographic factors. These environmental aspects interfere directly with lifestyle habits adopted and are decisive in the maintenance of health, since they influence opportunities of access to adequate environments for the practice of physical activity, health information and services, as well as the availability of food in the household and access to technologies, among others.^29^ These environmental factors, therefore, also directly impacts in the choice of way of commuting, since it involves issues of accessibility and safety, which take into account aspects such as access to streets and sidewalks, lighting and safety on the way; public policies related to this issue should be encouraged.^26^ It is important to highlight some of the strengths of the present study, such as the sample, which represents the school population of the municipality. We also highlight the fact that the study was conducted and provided data from the population of a city in the countryside of the state, differing from most investigations conducted in capitals and large centers. This study is still innovative as it assesses the association between commuting to school and several cardiometabolic risk factors in the Brazilian population of children and adolescents, as no other studies analyzing the same variables have been found. Our findings contribute to the progress of knowledge in this area.

As weaknesses and limitations of the research, we highlight the fact that the way of commuting of the students was investigated through a self-referenced questionnaire, and also the fact that the analysis does not consider distance traveled or time spent in commuting. Also, as this is a cross-sectional study, it is not possible to determine a cause-and-effect association between variables, once it is not possible to determine the temporality of the events studied. Other physical and leisure activities practiced by schoolchildren have not been considered, and may also influence the parameters evaluated, since those who passively commute may have higher levels of physical activity or similar to those who actively commute to school, directly influencing their health parameters. However, a study with Portuguese schoolchildren adjusted the variable physical activity through accelerometry and observed that, although 75% of the sample commuted actively, 85% of schoolchildren were not sufficiently active. Among those who actively commuted to school, 86% took less than 15 minutes to get to school and only 13% took more than 15 minutes. Still, the results suggest that walking to school results in better WC and HDL levels, and may indicate that even short walks can play an important role in health.^4^

Conclusion is that the prevalence of active commuting among schoolchildren is low; less than half of the sample go to school on foot or by bicycle. Active commuting to school showed a crude association with glucose and LDL cholesterol levels in schoolchildren, so active commuting seems to help reduce these levels. However, sociodemographic variables seem to interfere with this association, so further studies investigating these associations in the school population are needed.
